# The EGFR-HSF1 axis accelerates the tumorigenesis of pancreatic cancer

**DOI:** 10.1186/s13046-020-01823-4

**Published:** 2021-01-09

**Authors:** Weikun Qian, Ke Chen, Tao Qin, Ying Xiao, Jie Li, Yangyang Yue, Cancan Zhou, Jiguang Ma, Wanxing Duan, Jianjun Lei, Liang Han, Li Li, Xin Shen, Zheng Wu, Qingyong Ma, Zheng Wang

**Affiliations:** 1 Department of Hepatobiliary Surgery, The First Affiliated Hospital of Xi’an Jiaotong University, 277 West Yanta Road, Xi’an, 710061 China; 2 Department of Gastrointestinal and Pancreatic Surgery, Zhejiang Provincial People’s Hospital of Hangzhou Medical College, Hangzhou, 310014 China; 3 Department of Anesthesiology, The First Affiliated Hospital of Xi’an Jiaotong University, Xi’an, 710061 China; 4 Department of Ophthalmology, The First Affiliated Hospital of Xi’an Jiaotong University, Xi’an, 710061 China

**Keywords:** Pancreatic ductal adenocarcinoma, Heat shock factor 1, Epidermal growth factor receptor, Transgenic mice, Tumorigenesis

## Abstract

**Background:**

Pancreatic ductal adenocarcinoma (PDAC) is one of the most malignant diseases because of its non-symptomatic tumorigenesis. We previous found heat shock factor 1 (HSF1) was critical for PDAC progression and the aim of this study was to clarified the mechanisms on early activation of HSF1 and its role in the pancreatic cancer tumorigenesis.

**Methods:**

The expression and location of HSF1 on human or mice pancreatic tissues were examined by immunohistochemically staining. We mainly used pancreatic acinar cell 3-dimensional (3D) culture and a spontaneous pancreatic precancerous lesion mouse model called *LSL-Kras*^*G12D/+*^*; Pdx1-Cre* (KC) (and pancreatitis models derived from KC mice) to explore the pro-tumorigenesis mechanisms of the HSF1 in vitro and in vivo. Bioinformatics and molecular experiments were used to explore the underlying mechanisms between HSF1 and epidermal growth factor receptor (EGFR).

**Results:**

In this study, we found that pharmacological inhibition of HSF1 slowed pancreatic cancer initiation and suppressed the pancreatitis-induced formation of pancreatic precancerous lesion. Next, bioinformatics analysis revealed the closely linked between HSF1 and EGFR pathway and we also confirmed their parallel activation in pancreatic precancerous lesions. Besides, the pharmacological inhibition of EGFR suppressed the initiation of pancreatic cancer and the activation of HSF1 in vivo. Indeed, we demonstrated that the EGFR activation that mediated pancreatic cancer tumorigenesis was partly HSF1-dependent in vitro.

**Conclusion:**

Hence, we concluded that the EGFR-HSF1 axis promoted the initiation of pancreatic cancer.

**Supplementary Information:**

The online version contains supplementary material available at 10.1186/s13046-020-01823-4.

## Background

Pancreatic ductal adenocarcinoma (PDAC) is a highly malignant digestive system tumor with a 5-year survival lower than 9% [1]. Because of the insidious initiation, pancreatic cancer patients are often diagnosed at an advanced stage when they cannot undergo radical resection; additionally, because of the rapid progression of pancreatic cancer, the clinical efficacy of these treatments remains poor [2, 3]. Taken together, exploring the mechanism of pancreatic cancer initiation is key to improving its poor prognosis.

Heat shock factor 1 (HSF1) and its downstream heat shock proteins (HSPs) mediated heat shock responses (HSRs) are important for maintain cellular proteostasis by assisting with the synthesis and degradation of bioactive proteins during heat shock stress (HSSs) and other proteotoxic stress (PTSs) [4, 5]. Mentionable, tumor tissues have been referred to as a “chronic PTSs” to the organism and cancer cells seems to be more susceptible to proteomic perturbation compared to normal cells, which suggested the core role of HSF1 on sustain the malignancy of cancer [6]. Consistently, studies have shown that HSF1 was continuously activated and its downstream HSP70/90 were obviously elevated in many cancers [7, 8], and we also found that the abnormal activation of HSF1 and its targets even in pancreatic intraepithelial neoplasias (PanINs), the most common precancerous lesion of pancreatic cancer, which indicates that HSF1 activation is an early event in pancreatic cancer and HSF1 may participates in the initiation of pancreatic cancer.

Epidermal growth factor receptor (EGFR) and its downstream pathways (such as Ras-MAPK/PI3K-AKT-mTOR) are involved in the tumorigenesis of many cancers, especially in *KRAS* oncogene mutant-driven cancers such as PDAC (at least 90% of PDAC patients harbor *KRAS* oncogene mutations and there are few effective strategies) [9, 10]. In addition, studies have shown that EGFR is essential for the formation of acinar-ductal metaplasia (ADM) and the development of PDAC [11]. As ADM is a necessary process for the malignant transformation of pancreatic acinar cells (into pancreatic precancerous lesions PanINs), it is reasonable to believe that EGFR and its downstream pathways play a critical role in the initiation of pancreatic cancer [12]. Indeed, Tang Z and colleagues proved that MEK, a classic downstream target of EGFR mediated MAPK pathway, is pivotal in maintaining the proteostasis of melanoma cells by phosphorylating and activating HSF1, which means that Ras-MEK-HSF1 and the upstream molecule EGFR may play an important role in the initiation and progression of melanoma [6]. However, the role of the EGFR-HSF1 axis in the tumorigenesis of pancreatic cancer needs to be investigated.

In this study, by using a transgenic mouse model that can mimic the whole developmental process (from normal acinar cells to invasive PDAC) of pancreatic cancer called *LSL-Kras*^*G12D/+*^*; Pdx1-Cre* (KC) mice, we demonstrated that the EGFR-HSF1 axis promoted the initiation of pancreatic cancer.

## Methods

### Reagents

Cerulein (40 μg/kg or 5 μg/mouse), KRIBB11 (mainly 2 μM for cells and 50 mg/kg for mice), erlotinib (2 μM for cells and 100 mg/kg for mice), selumetinib (100 nM for cells) and torkinib (50 nM for cells) were purchased from MedChem Express (Monmouth Junction, NJ, USA). Recombinant human epidermal growth factor (EGF mainly 20 ng/ml for cells) and transforming growth factor alpha (mainly TGFα 50 ng/ml for cells) were purchased from PeproTech (Rocky Hill, NJ, USA). All reagents were stored according to the manufacturer’s instructions.

### Human tissue specimens obtain and cell culture

Human pancreatic cancer tissue specimens or normal pancreas tissue specimens were collected at the Department of Hepatobiliary Surgery, the First Affiliated Hospital of Xi’an Jiaotong University. The human BxPC-3, CFPAC-1, MIAPaCa-2, AsPC-1 and PANC-1 pancreatic cancer cell lines and pancreatic ductal progenitor cell line hTERT-HPNE (all obtained from the Chinese Academy of Sciences Cell Bank of Type Culture Collection, Shanghai, China) were cultured in appropriate culture medium (Gibco, Grand Island, NY, USA) with 10% (AsPC-1 20%) fetal bovine serum (FBS, HyClone, Logan, UT, USA) and 1% penicillin-streptomycin (Gibco) in a standard incubator with a 5% CO_2_ atmosphere at 37 °C. After appropriate grouping and intervention, we extracted the mRNA/protein from the cells for analysis. The study was conducted in accordance to the Declaration of Helsinki, and all protocols were approved by the Ethical Committee of the First Affiliated Hospital of Xi’an Jiaotong University, Xi’an, China.

### Genetically engineered mouse models (GEMMs)

The GEMMs of pancreatic cancer have been previously described by our group [13]. Briefly, we first purchased *LSL-Kras*^*G12D/+*^ (K) mice and *Pdx1-Cre* (C) mice from the Nanjing Biomedical Research Institute of Nanjing University, Nanjing, China. Then, we crossed them to generate *LSL-Kras*^*G12D/+*^*; Pdx1-Cre* (KC) mice which accurately recapitulate the whole process, from the initiation to the progression of human pancreatic cancer (Additional file 1: Fig. S1A and D). The study protocols were approved by the Ethical Committee of the First Affiliated Hospital of Xi’an Jiaotong University, Xi’an, China.

### In vivo therapeutic strategy and tissue preparation

As we mentioned previously, we treated KC mice with a series of strategies to research the initiation of pancreatic cancer [14]. (All the intervention groups included 6 randomly assigned KC mice, start at 8 weeks of age):

For the initiation assay, we treated KC mice with KRIBB11 (50 mg/kg/day, intraperitoneal injection, for 1 month), erlotinib (100 mg/kg/day, gavage, for 1 month) and used 0.9% NaCl as the corresponding vehicle (Additional file 1: Fig. S1C left).

For the in vivo ADM formation assay, we first treated KC mice with cerulein (40 μg/kg, 6 times a day, for 2 days; and recovery for another 5 days) to generate an acute pancreatitis-ADM models (AP-ADMs). During this period, these mice were also undergoing KRIBB11 or 0.9% NaCl therapy (Additional file 1: Fig. S1C middle).

For the in vivo PanINs formation assay, we first treated KC mice with cerulein (5 μg/mouse, 5 times per week, for 2 weeks; and recovery for another 2 weeks) to generate a chronic pancreatitis-PanINs model (CP-PanINs). During this period, these mice were also undergoing KRIBB11 or 0.9% NaCl therapy (Additional file 1: Fig. S1C right).

After euthanasia, we harvested mouse pancreas tissues and measured their weights. Then, the pancreas was immediately fixed in 10% buffered formalin (and embedded in paraffin) or in liquid nitrogen (and stored at − 80 °C).

### Histopathological analysis

As previously described [13], to observe the pathological morphology of the KC mouse pancreas, Hematoxylin and Eosin (H&E) staining and Masson’s staining of collagen (blue+ area) were performed using a kit from Sigma-Aldrich (St. Louis, MO, USA) according to the manufacturer’s instructions to examine the stromal elements of the KC mouse pancreas. Then, immunohistochemical (IHC) staining and double-label immunofluorescence (IF) staining was performed according to the manufacturer’s instructions. Primary antibodies were used against the following antigens: Ki67 (Servicebio, Wuhan, Hubei, China), HSP70 and HSP90 (Proteintech Group, Chicago, IL, USA), S326 p-HSF1 (Bioss, Woburn, MA, USA) and HSF1, Amylase, CK19, EGFR, Y1068 p-EGFR (Abcam, Cambridge, MA, USA). Five fields (400×) were randomly selected from each slide, and the proportion of the positive area in each field was determined using a software ImageJ (version 1.52a, National Institutes of Health, Bethesda, MD, USA).

### Pancreatic acinar cell 3-dimensional (3D) culture

We performed pancreatic acinar cell 3D culture assays based on a protocol that was previously reported [15]. Briefly, we first harvested the pancreas from 4-week-old KC/C mice. After 2–3 washes, the pancreas was chopped into small pieces in 4 °C hanks balanced salt solution (HBSS, Thermo Fisher Scientific, Waltham, MA, USA) and then transferred to 5 ml of 4 °C 5% FBS-HBSS containing 1 mg collagenase P (Roche, Basel, Switzerland). All these operations were performed on ice. Next, the pancreas pieces were shaken in a 37 °C shaker for approximately 20 min to obtain a cell suspension (closely monitored during this period and stopped shaking when most of the tissue clumps were gone and the suspension looked cloudy). Then, the cell suspension was washed and resuspended in 4 °C 5% FBS-HBSS, centrifuged (1500 rpm, 2 min) three times and filtered through 500 μm and 100 μm mesh filters. Then, filtered cell suspension was mixed into 4 °C 30% FBS-HBSS and was centrifuged at 1000 rpm for 2 min. Finally, the cell pellet was resuspended in 8–10 ml 3D culture medium to seed individual acini clusters. The 3D culture medium (100 ml) contained 1 ml FBS, 1 ml penicillin-streptomycin, and 10 mg soybean trypsin inhibitor (Sigma-Aldrich) and 100 μg dexamethasone (Sigma-Aldrich) in 100 ml RPMI-1640 (Gibco). For 3D culture, equal parts of the cell suspension and collagen mix (9-parts rat tail collagen I plus 1-part 10× 3D culture medium and 100 μl 4.2% NaHCO_3_) were mixed and plated into 24-well tissue culture plates, which contained 250 μl collagen mix in the bottom of each well. Then, 3D culture medium was added after the mix had solidified and the medium was changed on days 1 and 3 (KC mice acinar or C mice acinar treated with TGFα will often form the ductal-like sphere (ADM structure in vitro) as early as day 1 and 90% in day 5 [15]), so we observed the sphere formation on day 0, 1, 3 and day 5 (Additional file 1: Fig. S1B) and the sphere diameter/number was counted on day 5 (5 fields/well, 6 wells/group). For mRNA and protein extraction, we used the appropriate kits and followed the manufacturer’s instructions.

### Quantitative real-time PCR (qRT-PCR)

qRT-PCR experiments were performed as described previously [13]. The PCR primer sequences (mouse) that were used were as follows: Amylase (Amy2), forward TTGCCAAGGAATGTGAGCGAT, reverse CCAAGGTCTTGATGGGTTATGAA; CK19 (Krt19), forward GTTCAGTACGCATTGGGTCAG, reverse GAGGACGAGGTCACGAAGC; HSP70 (Hspa1a), forward TGGTGCAGTCCGACATGAAG, reverse GCTGAGAGTCGTTGAAGTAGGC; β-actin (Actb)**,** forward GTGACGTTGACATCCGTAAA, reverse TAAAACGCAGCTCAGTAACA. A Prime Script RT reagent kit (TaKaRa, Dalian, China) was used to reverse transcribe the total RNA into cDNA. Real-time PCR was conducted using a CFX Manager 2.1 fluorescent quantitative PCR kit (Bio-Rad Laboratories, Hercules, CA, USA). β-actin was used as an internal control.

### Western blot assays

Western blot experiments were performed as described previously [13]. Briefly, total protein from cells was prepared according to the manufacturer’s instructions. After protein concentration determination and gel electrophoresis, the proteins were transferred onto polyvinylidene difluoride membranes, and then the membranes were blocked in 10% nonfat dry milk powder (dissolved in PBS containing 0.1% Tween-20, PBST) for 2 h and subsequently incubated with primary antibodies overnight at 4 °C. The primary antibodies against HSP70 and β-actin that were used in this study were from Proteintech Group. The primary antibodies against S326 p-HSF1 was obtained from Bioss, the primary antibodies against HSF1, Amylase, CK19, EGFR and Y1068 p-EGFR were purchased from Abcam. The primary antibody against PCNA was purchased from Santa Cruz Biotechnology (Santa Cruz, CA, USA). Following incubation with secondary HRP-coupled antibodies for 1 h at room temperature, the membranes were washed with PBST, and the immunocomplexes were detected using an enhanced chemiluminescence kit and a Molecular Imager ChemiDoc XRS System (Bio-Rad Laboratories). β-actin was used as an internal control.

### Bioinformatics analysis

Two online bioinformatics analysis websites (GEPIA, http://gepia.cancer-pku.cn/, and Kaplan-Meier Plotter, http://www.kmplot.com) were used to analyze: 1) the relationship between mRNA level of HSF1 and pancreatic cancer patients’ prognosis; 2) the differential expression of HSF1 target genes in pancreatic cancer and normal pancreas tissues; 3) the relationship between EGFR pathway molecules and HSF1/HSF1 target genes.

Besides, to further analyses the possible functions of HSF1, we conducted a batch correlation analysis of HSF1 related molecules and the online software “the Database for Annotation, Visualization and Integrated Discovery” (DAVID, https://david.ncifcrf.gov/) were used to perform Gene Ontology (GO) analysis and Kyoto Encyclopedia of Genes and Genomes (KEGG) pathway enrichment analysis. *P* < 0.05 and counts > 2 were set as the threshold values. R software (version 3.6.3, https://www.r-project.org/) were used to draw diagram.

Indeed, to elucidate the effect of the certain single gene on the clinical and biological characteristics of pancreatic cancer, we firstly obtained a “The Cancer Genome Atlas (TCGA)” dataset corresponding to 182 pancreatic cancers patients’ tissues (4 normal pancreas tissues and 178 tumor tissues) from the TCGA database (https://portal.gdc.cancer.gov/); besides, a “Genotype-Tissue Expression (GTEx)” dataset corresponding to 167 normal pancreas tissues was obtained from GTEx database (https://www.gtexportal.org/). Next, the R language package [16, 17] limma, beeswarm, survival and survminer were used to: 1) merge the two datasets (171 normal vs 178 tumor), 2) extract the different expression of the certain single gene in normal tissues vs tumor tissues, 3) analyze the relationship between the certain single gene and survival or other clinical characteristics of pancreatic cancer patients (high- and low-expression groups were defined by median values of samples), 4) build univariate and multivariate COX regression models towards the certain single gene in pancreatic cancer. Finally, the biological pathways and transcription factors’ binding motifs/transcription factors’ signature or target gen sets [18] related to the certain single gene (according to above TCGA datasets) and to the differentially expressed gene set GSE98399 (created by Bauer TW and colleagues [19], according to https://www.ncbi.nlm.nih.gov/geo/, the mRNA expression profiles of acute vehicle control treatment group and acute trametinib (a MEK inhibitor) plus lapatinib (an EGFR inhibitor) treatment group) were evaluated by using “Gene Set Enrichment Analysis software (GSEA)” [20], and the number of permutations was set at 1000. Enrichment results satisfying FDR q-value < 0.25 or NOM *p*-value < 0.05 were regarded statistically significant. R software were used to draw diagram.

### Statistical analysis

The data are presented as the mean ± SD. Using the GraphPad Prism software package (GraphPad Prism version 6.0; La Jolla, CA, USA), the differences in the in vivo data were assessed with a Student’s t-test or one/two-way ANOVA. All tests were two-sided, and a *P* value < 0.05 was considered statistically significant.

## Results

### Abnormal HSF1 activation is an early molecular event in pancreatic cancer tumorigenesis

**Fig. 1:**
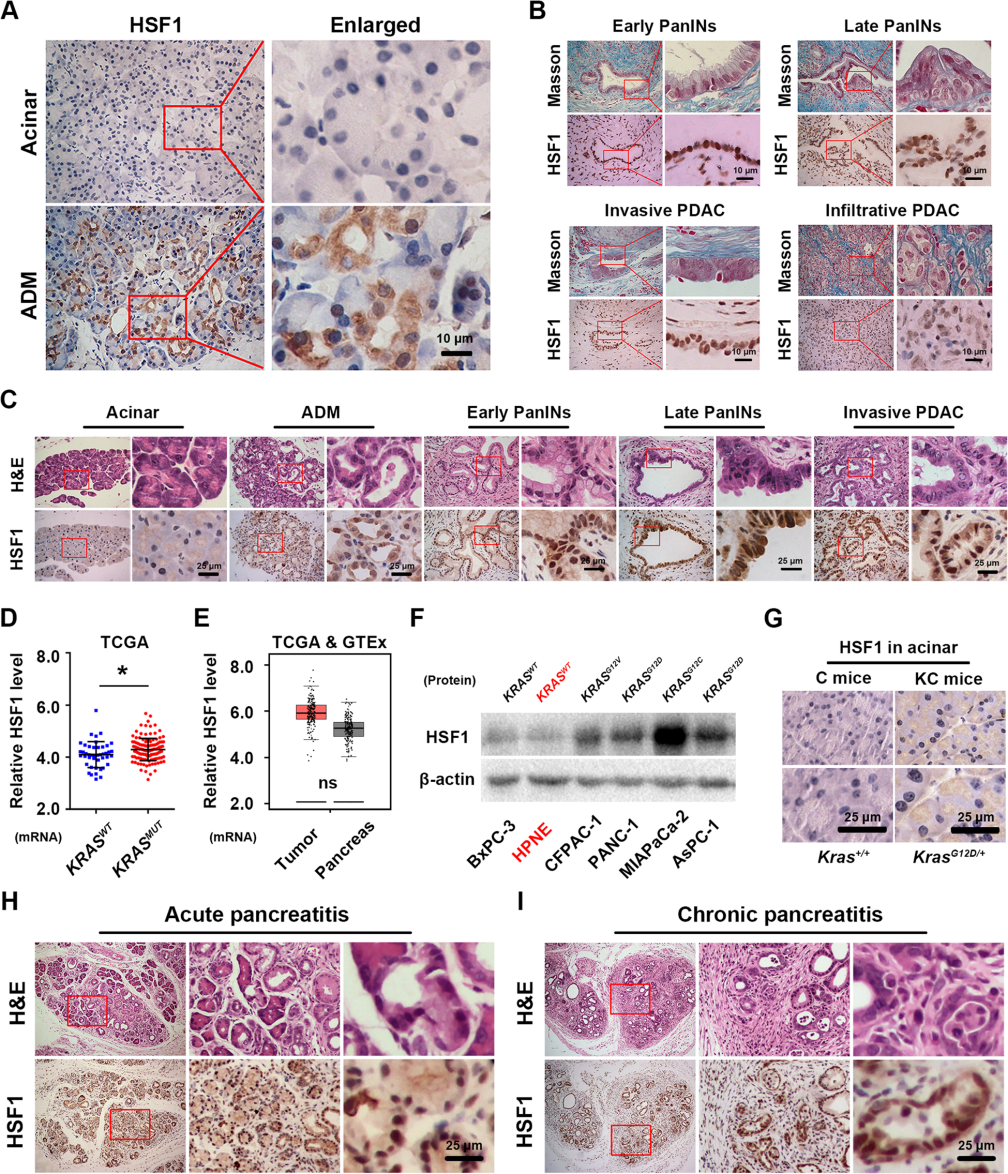
Abnormal HSF1 activation is an early molecular event in pancreatic cancer tumorigenesis. **a-b** Representative IHC staining of HSF1 positive cells and Masson staining of collagen I positive fibrosis in all stages of in all stages of the tumorigenesis of human pancreatic cancer (normal acinar and ADM (**a**), early PanINs, late PanINs, invasive and infiltrative PDAC tissues (**b**). **c** Representative histopathology (H&E staining) and the IHC staining of HSF1-positive cells in all stages of the tumorigenesis of murine (KC mice) pancreatic cancer. **d** The relative HSF1 expression in pancreatic cancer tissues of mutant *KRAS* samples (*KRAS*^*MUT*^) and non-mutant *KRAS* samples (*KRAS*^*WT*^) samples according to TCGA datasets. **e** The relative HSF1 expression in normal pancreas tissues and pancreatic cancer tissues according to TCGA & GTEx datasets. **f** The relative HSF1 protein expression in pancreatic ductal progenitor cell line hTERT-HPNE and pancreatic cancer cell lines BxPC-3, CFPAC-1, PANC-1, MIAPaCa-2, and AsPC-1. **g** Representative IHC staining of HSF1-positive cells in pancreatic acinar cells from C mice and KC mice pancreatic cancer. **h-i** Representative histopathology (H&E staining) and IHC staining of HSF1-positive cells in the acute pancreatitis tissues (**h**) and chronic pancreatitis tissues (**i**) of KC mice. Scale bars = 10/25 μm. * *P* < 0.05, ns *P* > 0.05

As in our previous study, activated HSF1 level was elevated in human/mice PDAC and was associated with it progression [13]. In this study, compared to normal pancreatic acinar, we found that the starting significant accumulation of HSF1 in the cytoplasmic of human pancreatic ADM structure (Fig. 1a); however, these activated HSF1 were quickly translocated into nucleus of precancerous lesions even as the in early PanINs formation and lasted until invasive PDAC stage, along with an abundant desmoplastic reaction (Fig. 1b), which means HSF1 and its activation may participate in the initiation of human pancreatic cancer. Next, by using GEPIA and Kaplan-Meier Plotter, we found that HSF1 mRNA level has limited correlation to poor prognosis (overall survival (OS) and disease free survival (DFS)) of pancreatic cancer patients (Additional file 1: Fig. S1F-H), and HSF1 target genes, such as HSPA1A/HSP90AA1, were elevated in pancreatic cancer compared to in the normal pancreas (Additional file 1: Fig. S1I and J). Besides, we also found there was no relationship between the HSF1 expression and the clinical characteristics of pancreatic cancer patients such as tumor stage (Additional file 1: Fig. S1K and Additional file 2: Table S1), and HSF1 was not an independent risk factors for OS of pancreatic cancer patients (Additional file 1: Fig. S1L and Additional file 2: Table S1) according to TCGA datasets. To sum up, all the findings suggested that the central roles of HSF1 may mainly enrichment in the initiation of human pancreatic cancer rather than its progression.

Besides, by using the well tumorigenesis model of pancreatic cancer called KC mice (which harbor a pancreas specific mutant *Kras*^*G12D*^ oncogene), we found the same phenomenon that there was little HSF1 in normal acinar; however, the accumulation of HSF1 was founded in murine ADM cytoplasmic and in the nucleus of precancerous lesions/invasive PDAC tissues (Fig. 1c). Indeed, we next want to investigate whether the HSF1 expression was correlated with *KRAS* oncogene mutation. In mRNA level, according to TCGA datasets, pancreatic cancer samples which harbored mutant *KRAS* have a higher level of HSF1 compared to no mutation samples (Fig. 1d and Additional file 2: Table S1). However, we also found there have no significant difference between normal pancreas and pancreatic tumor tissues in mRNA level (Fig. 1e). Hence, to further illustrate above doubt, in protein level, we found that compared to pancreatic ductal progenitor cell line hTERT-HPNE (which harbor *KRAS*^*WT*^ and can reflect the properties of intermediary cells produced during ADM [21, 22]), pancreatic cancer cell lines harbor relative high expression of HSF1 (Fig. 1f). Mentionable, similarly to Liang W and colleagues findings [23], as for the difference of pancreatic cancer cell lines in Fig. 1f, we also detected that HSF1 expression in mutant *KRAS* cell lines [24] (AsPC-1/*KRAS*^*G12D*^, MIAPaCa-2/*KRAS*^*G12C*^, PANC-1*/KRAS*^*G12D*^, CFPAC-1/*KRAS*^*G12V*^) were relative higher than wild-type *KRAS* cell line [24] (BxPC-3/*KRAS*^*WT*^). Interestingly, we also demonstrated that in mice pancreas, C mice acinar cells (*Kras*^*WT*^) have scarcely any HSF1 expression but KC mice acinar cells (*Kras*^*G12D*^) have little HSF1 expression in their cytoplasm (Fig. 1g). As we all know, *KRAS* oncogene mutation was a switch of pancreatic cancer initiation [25], hence, above findings suggested that HSF1 may be a critical molecular for pancreatic cancer tumorigenesis again.

In addition, studies have proven that both acute and chronic pancreatitis are the key risk factors for pancreatic cancer [26–28], so we treated 8-week-old KC mice (when PanINs lesions starting formation, as shown in Additional file 1: Fig. S1E) with cerulein to induce pancreatitis, and we found that HSF1 was expressed at a high level and had translocated from the cytoplasm to the nucleus during the pancreatitis (Fig. 1h and i). In conclusion, abnormal HSF1 activation is an early molecular event in pancreatic cancer initiation and may participate its tumorigenesis.

### Pharmacological inhibition of HSF1 suppresses pancreatic cancer initiation

**Fig. 2:**
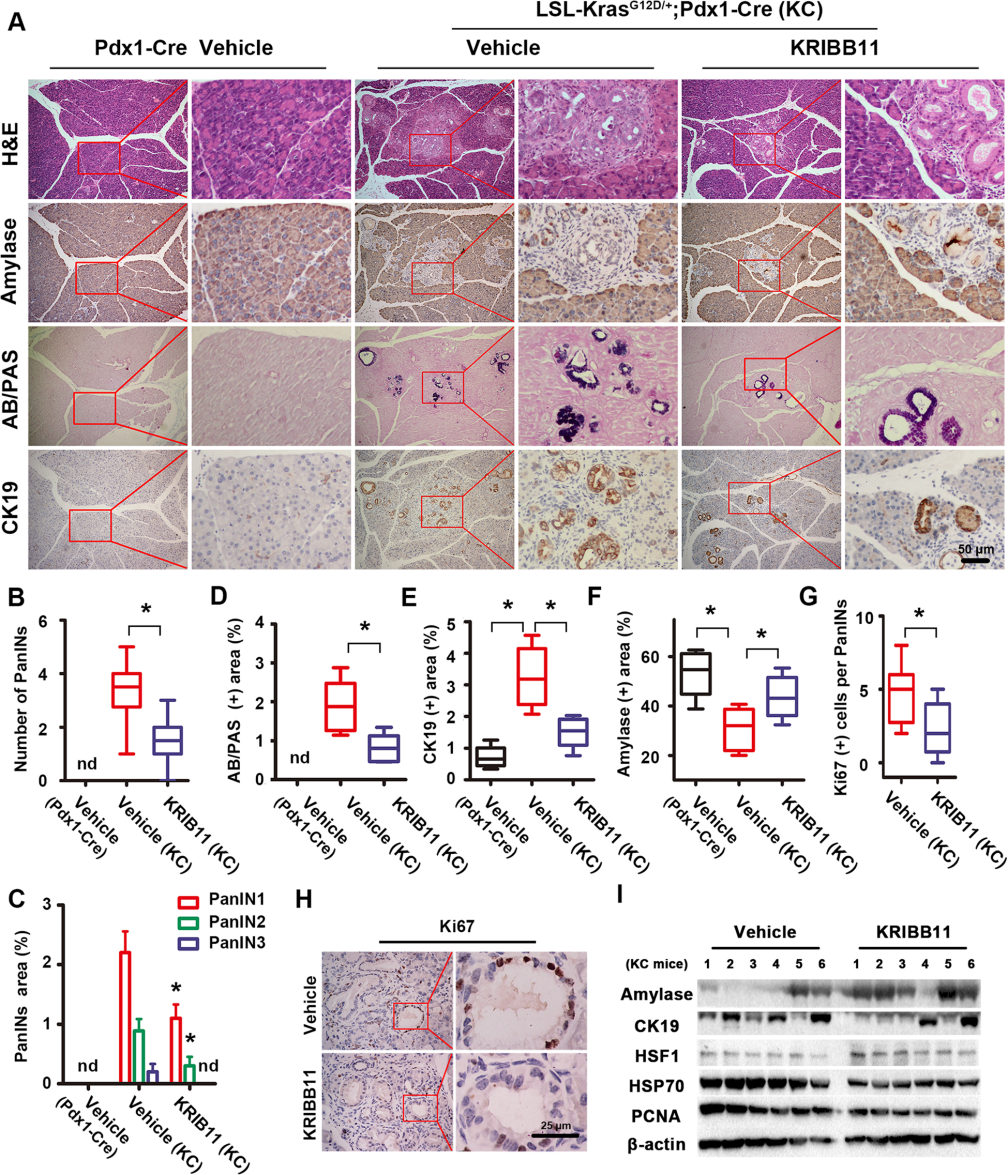
Pharmacological inhibition of HSF1 suppressed the initiation of pancreatic cancer. **a** Representative histopathology (H&E staining), low-grade PanINs lesions (AB/PAS staining) and the IHC staining of amylase (acinar marker), CK19 (ductal marker) in the pancreas of vehicle C mice, vehicle KC mice and KC mice treated with KRIBB11 (HSF1 inhibitor). **b-f** Quantification of the PanINs numbers (**b**) and the percentage of the PanINs area (**c**), the AB/PAS-positive blue area (**d**), the CK19-positive ductal area (**e**) and the amylase-positive acinar area (**f**) per 400× field in the pancreas of vehicle C mice, vehicle KC mice and KC mice treated with KRIBB11. **g-h** IHC staining and its quantification of Ki67-positive proliferative cells in PanINs structures per 400× field in the pancreas of vehicle KC mice and KC mice treated with KRIBB11. **i** Western blotting assay were performed to evaluate the effect of KRIBB11 on the expression of amylase, CK19, HSF1 and HSP70 and PCNA in KC mice pancreases. Scale bars = 25/50 μm. nd = not detected, * *P* < 0.05

To illuminate above hypothesis, we next treated KC mice with KRIBB11 (a well-known HSF1 inhibitor) to pharmacologically inhibit the activation of HSF1 (Additional file 1: Fig. S1C left), and we found that KRIBB11 reduced the area of pancreatic precancerous lesions (both the number and the area of PanINs at all stages) compared with that in vehicle KC mice (Fig. 2a-c). Along with this, we found that there were no low-grade pancreatic precancerous lesions (AB/PAS+ blue area) in vehicle C mice pancreases. However, vehicle KC mice had a number low-grade PanINs which indicated the initiation of pancreatic cancer; nevertheless, after treatment with KRIBB11, the AB/PAS+ blue area was obviously reduced (Fig. 2a and d), which demonstrates that HSF1 inhibition may suppress the formation of PanINs. Indeed, by using IHC staining for amylase (acinar marker) and CK19 (ductal marker), we found that KRIBB11 elevated the normal amylase+ acinar area and reduced the abnormal CK19+ ductal area in the KC mice pancreases compared to those of the control (Fig. 2a, e and f); consistently, by using western blotting assay, we found that the CK19 expression and the activation of HSF1 was inhibited by KRIBB11 (represented by the reduction of HSP70) in KC mice pancreas (Fig. 2i), but the amylase expression was upregulated in KRIBB11 treated KC mice compared with vehicle KC mice (Fig. 2i). All the findings indicated that HSF1 inhibition may suppress the initiation of pancreatic cancer. Notably, studies have proved that the growth-promoting effect of HSF1 was mainly due to its regulatory activity for proliferation and metabolism [7], and we found that KRIBB11 treatment lead to a reduced number of Ki67+ proliferating cells in PanINs compared to that in the vehicles (Fig. 2g and h). Taken together, HSF1 plays a vital role in the initiation of pancreatic cancer.

**HSF1 is a critical participant in the fate change of pancreatic acinar cells.**

**Fig. 3:**
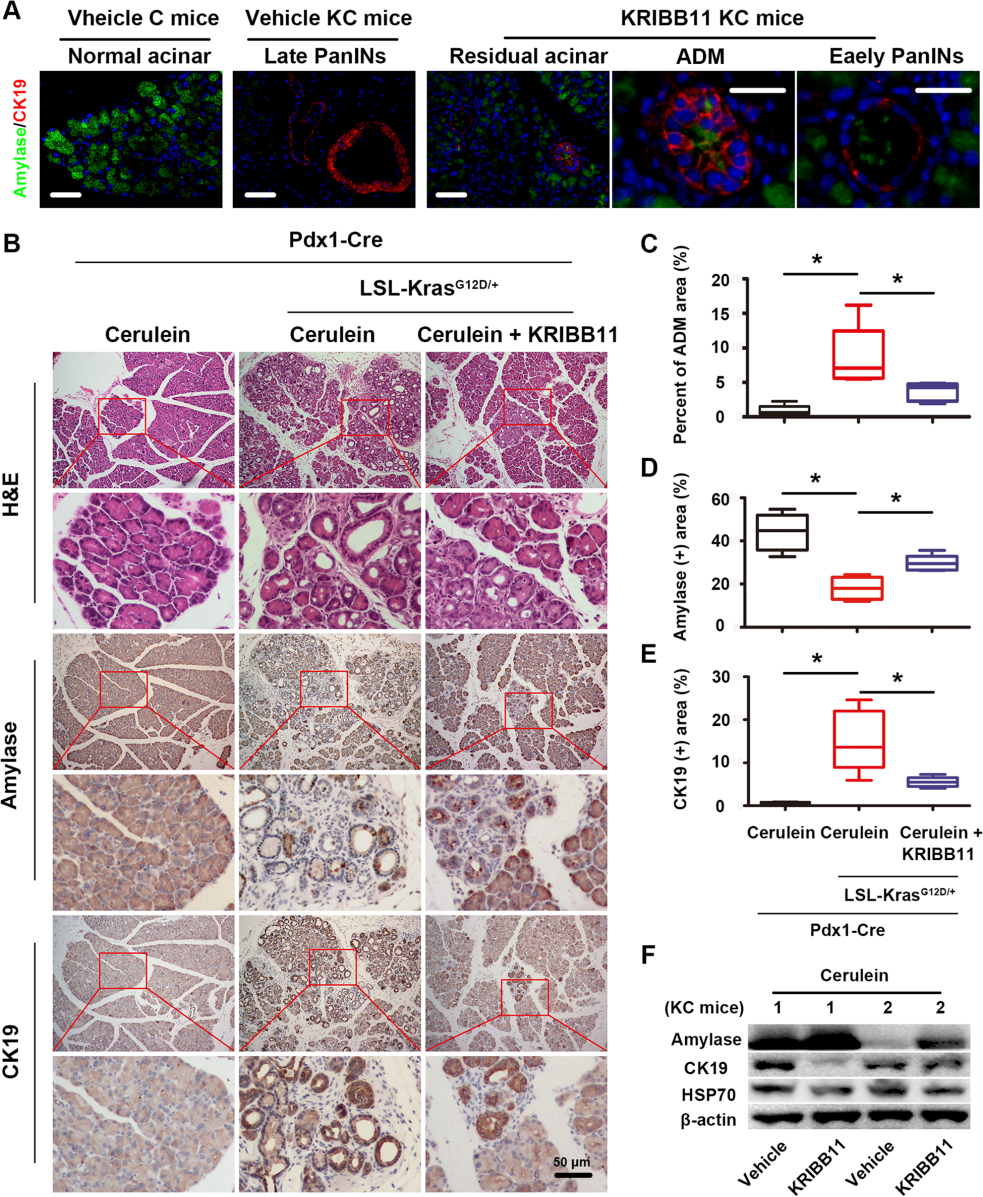
Pharmacological inhibition of HSF1 suppressed the formation of ADM in vivo. **a** Double-label IF staining showed the expression of amylase and CK19 in the pancreas of vehicle C mice, vehicle KC mice and KC mice treated with KRIBB11. **b** Representative histopathology (H&E staining) and the IHC staining of amylase (acinar marker), CK19 (ductal marker) in the pancreas of C mice treated with short-term cerulein, KC mice treated with short-term cerulein and KC mice treated with short-term cerulein plus KRIBB11. **c-e** Quantification of the PanINs area (**c**), the amylase-positive acinar area (**d**) and the CK19-positive ductal area (**e**) per 400× field in the pancreas of C mice treated with short-term cerulein, KC mice treated with short-term cerulein and KC mice treated with short-term cerulein plus KRIBB11. **f** Western blotting assay were performed to evaluate the effect of KRIBB11 on the expression of amylase, CK19 and HSP70 in KC mice (short-term cerulein) acute pancreatitis tissues. Scale bars = 50 μm. * *P* < 0.05

In the abovementioned initiation assay, we preliminarily illuminated that HSF1 might be related to the initiation of pancreatic cancer through pathological analysis. Next, we used double-label IF staining to verify these findings, and the results showed that some high-grade PanINs existed in vehicle KC mice pancreases (along with a loss of acinar structures); nevertheless, these effects were partly reversed after the inhibition of HSF1 (Fig. 3a). Specifically, compared to vehicle KC mice, KRIBB11 attenuated acinar loss; and interestingly, the precancerous lesions in the KRIBB11 group were mainly composed of ADM (both amylase+ and CK19+) and low-grade PanINs rather than high-grade PanINs (Fig. 3a), which insisted that HSF1 may participated in the fate change of pancreatic acinar cells. Hence, we used AP-ADMs (an in vivo ADM formation assay, see in Additional file 1: Fig. S1C middle) and pancreatic acinar cell 3D culture (an in vitro ADM formation assay, see in Additional file 1: Fig. S1B and Additional file 1: Fig. S2A) to further investigate this phenomenon in vivo and in vitro.

For the in vivo assay, after treating with cerulein for 2 days, both KC mice and C mice developed acute pancreatitis and raised lots of ADM (data not shown) in their pancreases. After 5 days of recovery, the pathologic morphology of the C mice pancreases had basically returned to a normal acinar structure (there were no ADM or PanINs in the pancreas, only the edema of acinar cells); however, the KC mice pancreases still exhibited a large amount of ADM and even PanINs, and this phenomenon was attenuated by treatment with KRIBB11 compared to that in the vehicles (Fig. 3b and c). Similarly, IHC staining showed that HSF1 inhibition increased the normal amylase+ acinar area and reduced the abnormal CK19+ ductal area in the KC mice pancreases (Fig. 1b, d and e). Consist of these findings, by using western blotting assay, we also found that KRIBB11 reversed the acinar loss and ductal gain which caused by short-term cerulein induced acute pancreatitis tissues (Fig. 3f). Synchronously, the expression HSP70 were also downregulated by KRIBB11 which insisted that the anti-ADM formation effect of KRIBB11 may related to its role on inhibiting HSF1 (Fig. 3f).

For the in vitro assay, we plated acinar cell clusters from KC/C mice in 3D conditions (Additional file 1: Fig. S2A) and treated them with KRIBB11 or not to explore the role of HSF1 on the initiation of pancreatic cancer in vitro. Before starting, we first excluded the toxic effect of KRIBB11 on KC mice acinar cells because primary pancreatic acinar cells were vulnerable even in 3D culture condition. The results showed that lower KRIBB11 consecration (≤ 10 μM) have no obvious toxic effect on the growth and ADM formation of acinar cells (Additional file 1: Fig. S2B, we observed the existence of phenotypic normal acinar (green), acinar with a phenotype that are undergoing ADM (blue) and ductal-like sphere (in vitro ADM structures, red) in the background of KRIBB11 2 μM after 3 days’ culture). Secondly, we treated acinar cells from C mice with KRIBB11 and found that HSF1 inhibition accelerated the death/necrosis and structural collapse of acinar cells (Additional file 1: Fig. S2C), which suggested that the core role of HSF1/HSPs on the protection of pancreas in stress condition [29]. Next, we treated acinar cells from KC mice with KRIBB11 and found that HSF1 inhibition suppressed the formation of ductal-like spheres, in other words, KRIBB11 inhibited the pancreatic cancer tumorigenesis in vitro (Additional file 1: Fig. S2D-F). By using qRT-PCR assay, we found that HSF1 inhibition reduced the expression of the ductal marker CK19 and HSP70; in contrast, KRIBB11 evaluated the expression of the acinar marker amylase (Additional file 1: Fig. S2G). Besides, we also corresponding lentiviral expression system to knock-down/over-expression of HSF1 to repeat above experiments and came out the same phenomena (Additional file 1: Fig. S2H-K, though the expression of amylase has no statistical difference, there was still a trend). In conclusion, all in vivo and in vitro findings indicated that HSF1 is related to the formation of ADM, in other words, HSF1 is a critical participant in the fate change of pancreatic acinar cells.

### HSF1 is a key molecule for the formation of pancreatic precancerous lesions

**Fig. 4:**
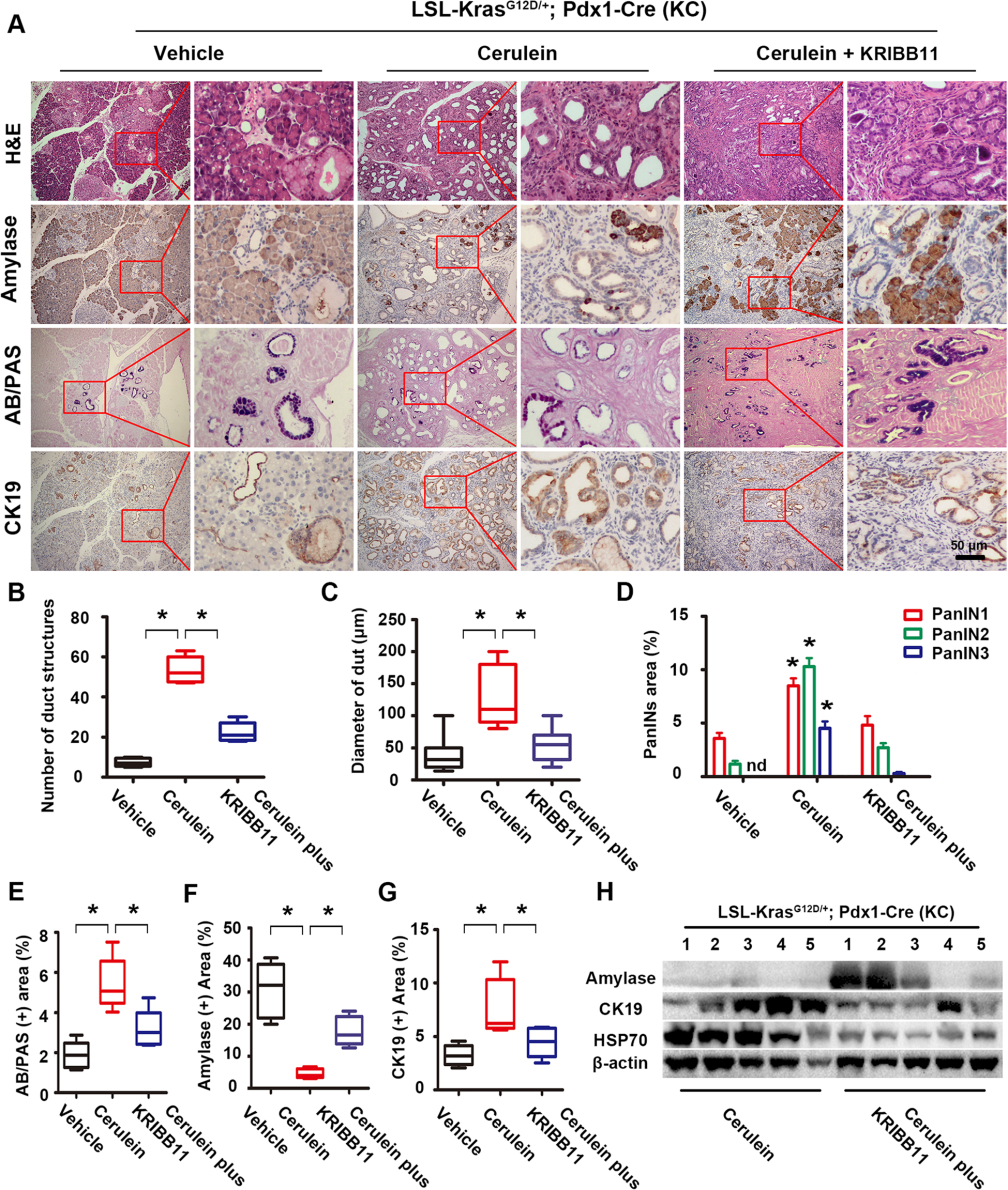
Pharmacological inhibition of HSF1 suppressed the formation of PanINs. **a** Representative histopathology (H&E staining), low-grade PanINs lesions (AB/PAS staining) and the IHC staining of amylase (acinar marker), CK19 (ductal marker) in the pancreas of vehicle KC mice, KC mice treated with long-term cerulein and KC mice treated with long-term cerulein plus KRIBB11. **b-c** Quantification of the number (**b**) and the diameter (**c**) of ductal-like structures per 400× field in the pancreas of vehicle KC mice, KC mice treated with long-term cerulein and KC mice treated with long-term cerulein plus KRIBB11. **d-g** Quantification of the percentage of the PanINs area (**d**), the AB/PAS-positive blue area (**e**), the amylase-positive acinar area (**f**) and the CK19-positive ductal area (**g**) per 400× field in the pancreas of vehicle KC mice, KC mice treated with long-term cerulein and KC mice treated with long-term cerulein plus KRIBB11. **h** Western blotting assay were performed to evaluate the effect of KRIBB11 on the expression of amylase, CK19 and HSP70 in KC mice (long-term cerulein) chronic pancreatitis tissues. Scale bars = 50 μm. nd = not detected, * *P* < 0.05

In the initiation assay described above, we preliminarily illuminated that HSF1 might be related to the formation of PanINs. Next, we used CP-PanINs (Additional file 1: Fig. S1C right) to further investigate this phenomenon in vivo. Compared with vehicle KC mice, the pathological morphology of the pancreases changed greatly in KC mice with chronic pancreatitis. Specifically, 12-week-old vehicle KC mice developed a certain amount of ADM and PanINs (mainly low-grade PanINs), and normal acinar structures still dominated the pancreas (Fig. 4a and d-f); however, in chronic pancreatitis KC mice, few normal acinar structures existed, and numerous abnormal ductal-like structures (predominantly belonging to high-grade PanINs and even local PDAC tissues, Fig. 4a-d and g) were present in the mice pancreases, along with abundant desmoplastic reaction. These findings are consistent with previous findings that demonstrated that chronic pancreatitis accelerates the development of pancreatic cancer [30]. Notably, the inhibition of HSF1 partially inhibited these processes; specifically, KRIBB11 reduced the loss of acinar cells/low-grade PanINs (Fig. 4a and d-f) and inhibited the formation of high-grade PanINs (Fig. 4a-d and g). Consist of these findings, by using western blotting assay, we also found that KRIBB11 reversed the acinar loss and ductal gain which caused by long-term cerulein induced chronic pancreatitis tissues (Fig. 4h). Synchronously, the expression HSP70 were also deregulated by KRIBB11 which insisted that the anti-PanINs formation of KRIBB11 may due to its HSF1 inhibition effect (Fig. 4h). To sum up, these phenomena demonstrated that HSF1 is a key molecule for the formation of pancreatic precancerous lesions.

### HSF1 is a potential downstream molecule of EGFR in pancreatic cancer tumorigenesis

**Fig. 5:**
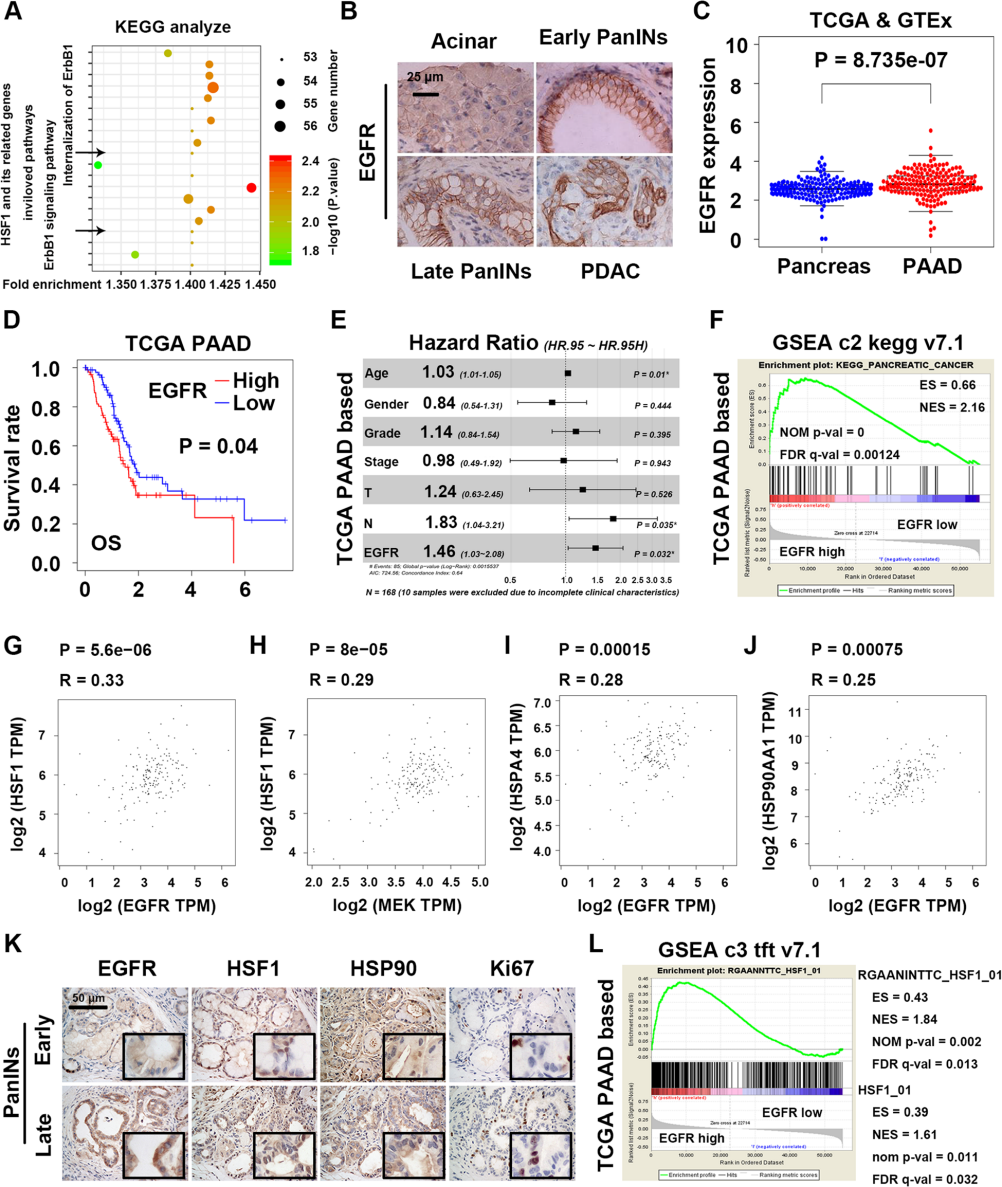
HSF1 was potential downstream molecule of EGFR in pancreatic cancer tumorigenesis. **a** The possible related biological pathways of HSF1 and its related proteins in pancreatic cancer. **b** IHC staining of EGFR in all stages of the tumorigenesis of pancreatic cancer in KC mice. **c** The relative EGFR expression in normal pancreas tissues and pancreatic cancer tissues according to TCGA & GTEx datasets. **d** The relationship between EGFR and the OS of pancreatic cancer according to TCGA database. **e** The forest graph showed HR and its 95% CI of EGFR in pancreatic cancer patients’ OS according to TCGA database. (**f**) GSEA analysis of biological pathways related to pancreatic cancer in EGFR high expression group vs EGFR low expression group according to TCGA database. **g-h** The relationship between HSF1 and EGFR singling pathway related molecules (EGFR (**g**)/MEK (**h**)) in pancreatic cancer. **i-j** The relationship between EGFR and HSF1 target genes (HSPA4 (**i**)/HSP90AA1(**j**)) in pancreatic cancer. **k** IHC staining of EGFR, HSF1, HSP90 and Ki67-positive areas in early/late PanINs of KC mice. **l** GSEA analysis of HSF1 binding motifs/signature (target genes gene sets, represented by RGAANNTTC_V$HSF1_01 and HSF1_01) in EGFR high expression group vs EGFR low expression group according to a TCGA datasets of pancreatic cancer. Scale bars = 25/50 μm. * *P* < 0.05

Through the above experiments, we found that HSF1 a key molecule in the initiation of pancreatic cancer. Next, to further analyze the possible molecular functions and biological pathways of HSF1 and its related proteins, we adopted DAVID to perform GO/KEGG analysis and the result showed that core relationship between HSF1/HSF1 related proteins and EGFR signaling (Fig. 5a and Additional file 2: Table S1). Besides, studies have shown that EGFR and its downstream pathways are involved in the tumorigenesis of many cancers (such as Ras-MEK-HSF1 axis regulates the development of melanoma [6]). So, we hypothesized that there may have an interaction between EGFR pathway and HSF1 in PDAC initiation. Firstly, we found that both pancreatic precancerous lesions and invasive PDAC harbored high levels of EGFR compared with normal pancreas (Fig. 5b), and this phenomenon was confirmed by TCGA datasets of pancreatic cancer (Fig. 5c). Interestingly, according to this datasets and Kaplan-Meier Plotter, we found high expression of EGFR was correlated with poor prognosis of pancreatic cancer patients (Fig. 5d and Additional file 3 Fig. S3A and B); besides, we found that EGFR expression was no obvious difference among pancreatic cancer patients with different clinical characteristics (such as age, clinical stage, pathological grade and lymph node metastasis status, Additional file 3: Table S2); however, we also found EGFR level, age and lymph node metastasis status were independent risk factors for OS of pancreatic cancer patients in the TCGA datasets (Fig. 5e). Indeed, GSEA analysis based on above TCGA datasets also revealed the core role of EGFR in pancreatic cancer related pathways and other well-known tumorigenesis related pathways (Fig. 5f and Additional file 3: Table S2). In conclusion, EGFR was important for pancreatic cancer initiation and progression.

However, whether EGFR pathway and HSF1 interact in pancreatic cancer and their “boss-subordinate relationship” remains unclear. Hence, to illuminate the above ambiguous relationship, firstly, by using GEPIA websites, we found EGFR pathway related molecules such as EGFR and MEK were correlated with HSF1 and its target genes (Fig. 5g-j) such as HSPA4 (HSP70) and HSP90AA1 (HSP90); next, above co-expression relation were confirmed by the IHC staining results that EGFR, HSF1 and HSP90 increased in parallel during the disease progression (Fig. 5k); indeed, by using GSEA analysis (based on TCGA datasets), we found HSF1 binding motifs/signature (target genes gene sets, represented by RGAANNTTC_V$HSF1_01 and HSF1_01) were enriched in group with high EGFR expression (Fig. 5l and Additional file 3: Table S2), which means EGFR pathway may has a positive regulating effect on HSF1. In conclusion, all the findings indicated that there was a correlation between EGFR and HSF1 in the tumorigenesis of pancreatic cancer. Interestingly, another GSEA analysis (based on GSE98399) also revealed that the acute inhibition of EGFR plus MEK caused the suppression of HSF1 binding motifs/signature as we mentioned before in a certain degree (Additional file 1: Fig. S3C and Additional file 4: Table S3, although NOM *p*-value > 0.05, it still has the expected trend). Combined with the preceding results, we concluded that EGFR may be the upstream molecular of HSF1.

### EGFR-HSF1 axis is momentous in pancreatic cancer initiation

**Fig. 6:**
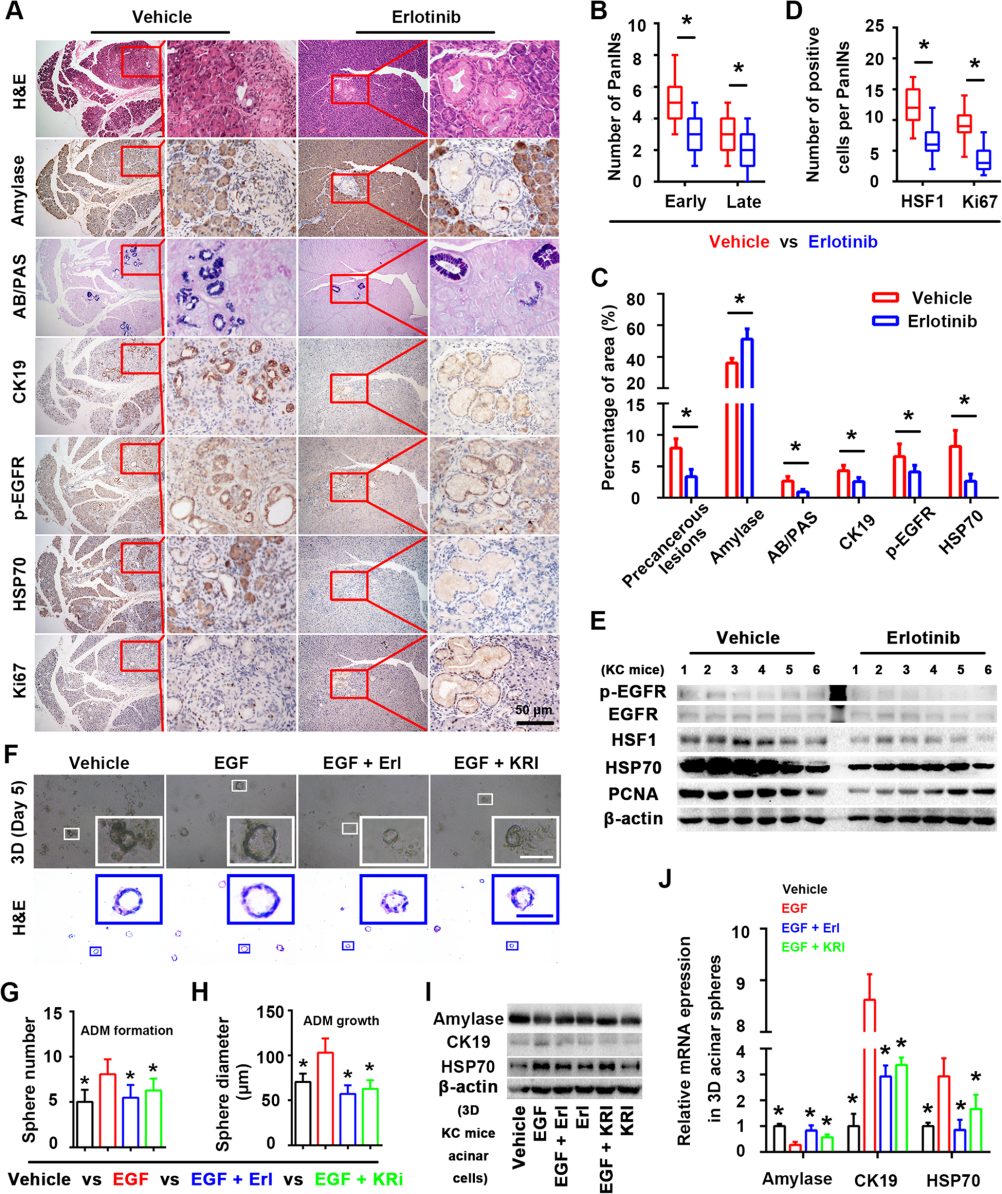
EGFR-HSF1 axis is momentous in pancreatic cancer initiation. **a** Representative histopathology (H&E staining), low-grade PanINs lesions (AB/PAS staining) and the IHC staining of amylase (acinar marker), CK19 (ductal marker), p-EGFR, HSP70 and Ki67 in the pancreas of vehicle KC mice and KC mice treated with erlotinib. **b** Quantification of the number of early and late PanINs per 400× field in the pancreas of vehicle KC mice and KC mice treated with erlotinib. **c** Quantification of the percentage of the precancerous lesion area, the AB/PAS-positive blue area, the amylase-positive acinar area, the CK19-positive ductal area, the p-EGFR-positive area and HSP70-positive area per 400× field in the pancreas of vehicle KC mice and KC mice treated with erlotinib. **d** Quantification of the number of HSF1- and Ki67-positive cells per 400× field in the pancreas of vehicle KC mice and KC mice treated with erlotinib. **e** Western blotting assay were performed to evaluate the effect of erlotinib on the expression of p-EGFR, EGFR, HSF1, HSP70 and PCNA in KC mice pancreas tissues. **f** Representative bright field/H&E staining images of 3D acinar/ductal-like spheres in the vehicle, EGF, EGF plus erlotinib (Erl) and EGF plus KRIBB11 (KRI) groups. **g-h** Quantification of the sphere number (**g**) and diameter (**h**) per 100× field in the vehicle, EGF, EGF plus erlotinib and EGF plus KRIBB11 groups (the EGF group was a control). **i** Western blotting was performed to evaluate the expression of acinar marker amylase, ductal marker CK19 and HSF1 target gene HSP70 among vehicle, EGF, EGF plus erlotinib, erlotinib, EGF plus KRIBB11 and KRIBB11 group in 3D acinar/ductal-like spheres. **j** Relative amylase, CK19 and HSP70 mRNA levels of 3D acinar/ductal-like spheres among the vehicle, EGF, EGF pluserlotinib and EGF plus KRIBB11 groups (the EGF group was a control). Scale bars = 50 μm. * *P* < 0.05

To verified above assumption, we treated KC mice with erlotinib (an inhibitor of EGFR) for 1 month and found that erlotinib suppressed the formation of pancreatic precancerous lesions (Fig. 6a-c), reduced the loss of amylase+ acinar structures, the gain of CK19+ ductal structures and the expression of HSF1 target gene HSP70 (Fig. 6a and c). Besides, EGFR inhibition suppressed the activation of HSF1 and the Ki67+ proliferation of cells in PanINs (Fig. 6a and d). Consist of these findings, western blotting assay also showed erlotinib inhibited the activation of EGFR (represented by the desecration of p-EGFR) and HSF1 (represented by the desecration of HSP70), and reduced the proliferation marker PCNA (Fig. 6e), which revealed the core role of EGFR on modulating pancreatic cancer initiation and HSF1 activation in vivo. However, does EGFR mediated pancreatic cancer initiation was HSF1 dependent remains unclear. Next, we adopted pancreatic acinar cell 3D culture to determine the effect of EGFR-HSF1 axis on the initiation of pancreatic cancer in vitro. We treated acinar cell clusters from KC mice with EGF (a natural activate legend of EGFR) and erlotinib/KRIBB11 and the results showed that EGFR activation significantly increased the number (ADM formation) and diameter (ADM growth) of ductal-like spheres, and this “sphere/ADM-promoting” ability was suppressed by erlotinib and KRIBB11 (Fig. 6f-h). Besides, by extracting acinar protein/mRNA, we found EGFR activation increased the expression of the ductal marker CK19 and HSF1 target gene HSP70, on the contrary, reduced the expression of the acinar marker amylase compared to those in the vehicles; however, these effects were partly blocked by EGFR/HSF1 inhibition (Fig. 6i-j). In conclusion, all the findings above suggested that EGFR mediated HSF1 activation play a key role in pancreatic cancer initiation in vivo and in vitro.

### EGFR stimulation activated HSF1 doubly in pancreatic acinar cells

Above findings suggested that EGFR-HSF1 axis is important for pancreatic cancer initiation both in vivo and in vitro. However, the mechanisms of HSF1 abnormal activation during pancreatic cancer initiation and the role of EGFR in this process remains unclear. Hence, we next aim to clarify above phenomena. Above in vivo studies showed that EGFR inhibition reduced the production of HSF1 (Fig. 5e); similarly, in this term, we found that KC mice acinar cells which harbor high level EGFR also have more HSF1 production compared with EGFR low expression acinar in vivo (Additional file 1: Fig. S3D). For in vitro assay, we also found that EGF/TGFα (24 h intervention) evaluated the level of HSF1 in both KC (relative severer) and C (relative slight) mice pancreatic acinar cells (Additional file 1: Fig. S3E); besides, to further investigate this phenomenon was EGFR and downstream pathway dependent, we treated acinar cells form C/KC mice with erlotinib/selumetinib (MEK inhibitor) and the results showed that EGFR activation elevated the production of HSF1 which can be suppressed by EGFR-MAPK pathway inhibitors (Additional file 1: Fig. S3F). Mentionable, this phenomenon was severer in KC mice acinar cells than C mice acinar cells (consist with previous findings in Fig. 7B) and same as our findings before (Fig. 1f), KC mice (*Kras*^*G12D*^ mediated hyper-activation of Ras-MAPK pathway) harbor relative high level HSF1 expression in acinar cells compared to C mice (*Kras*^*WT*^ mediated normal-activation of Ras-MAPK pathway), to sum up, above results suggested that apart from the activity of EGFR, abnormal Ras-MAPK can also influence HSF1 via unknown mechanism. Interestingly, we also found that acute EGF stimulation (30 min) on KC mice pancreatic acinar cells promoted the phosphorylation (the most common activate form) and nuclear translocation of HSF1 Additional file 1: Fig. S3G), however, these effects were inhibited by selumetinib and mTOR inhibitor torkinib (Additional file 1: Fig. S3G). Similarly, we also found the same phenomenon that EGFR inhibition can suppress the phosphorylation and translocation of HSF1 in to acinar nuclear obviously in vivo (Additional file 1: Fig. S3H). In conclusion, EGFR stimulation activated HSF1 doubly (both in total and phosphorylation level) in pancreatic acinar cells.

### HSF1 acts as the sensor of EGFR-Ras-MAPK hyper-activation induced PTSs during pancreatic cancer tumorigenesis

Above findings suggested that EGFR and downstream Ras-MAPK pathway play a vital role in protein production of HSF1 in an unknown mechanism. Obviously, we first thought about whether the activation of EGFR pathway would increase protein production of HSF1 via promoting its mRNA transcription and the answer is no, detailly, 24 h’ intervention of EGF/TGFα on KC mice pancreatic acinar cells have no influence on its HSF1 mRNA expression (Additional file 1: Fig. S3I), which suggested that EGFR and its downstream pathways influence the protein level of HSF1 in a non-transcription way. Interestingly, we suggested that the protein level of HSF1 have more clinical significance in pancreatic cancer initiation and progression previously, and as researcher mutually agreed, HSF1 and its target HSPs were key effectors that induce HSRs to maintain cellular proteostasis by perceiving HSSs and other PTSs (Additional file 1: Fig. S3J). Hence, we hypothesized that the abnormal activation EGFR and its downstream pathways may induce PTSs in pancreatic acinar cells and the accumulation/activation of HSF1 in pancreatic cancer tumorigenesis may be a passive response of the cells against to increasing PTSs.

To elucidate this assumption primarily, we chose IRE1α/PERK (two stress-sensing components of unfolded protein reaction (UPR)) as the markers to reflect the level of PTSs indirectly [31, 32]. The GSEA analysis based on a TCGA PAAD datasets was carried out to found the influence of EGFR/KRAS/MEK/ERK mRNA expression on these two molecular mediated UPR and the results showed that high expression of EGFR-Ras-MAPK pathways have a positive enriched tendency on these two UPR pathway (Additional file 1: Fig. S3K-N and Additional file 3: Table S2); besides, another GSEA analysis based on GSE98399 showed that the inhibition of EGFR plus MEK can partly have opposite effect against to above phenomenon (Additional file 1: Fig. S3O and Additional file 4: Table S3, although NOM *p*-value > 0.05, it still has the expected trend), which means higher activation level of EGFR-Ras-MAPK pathways may induce more serious UPR reaction (symbol of PTSs). Indeed, to further demonstrate whether the induction of IRE1α/PERK mediated UPR response or PTSs in pancreatic cancer can active HSF1, similar before, we adopted a well-known gene set from GSEA database (specifically, in REACTOME subsets and named REGULATION_OF_HSF1_MEDIATED_HEAT_SHOCK_RESPONS) to reflect the HSF1 and analyzed the enrichment of this sets in the background of different IRE1α/PERK mRNA expression according to a TCGA PAAD datasets. The results showed that high expression of PERK can positively enrich the activity of HSF1 (Additional file 1: Fig. S3P and Additional file 3: Table S2), but the phenomenon was not obvious in IRE1α (Additional file 1: Fig. S3P and Additional file 3: Table S2). Hence, above findings suggested that EGFR and its downstream Ras-MAPK pathway may activate HSF1 via induce the PTSs of pancreatic acinar cells indirectly, in other words, HSF1 may acts as the sensor of EGFR-Ras-MAPK hyper-activation induced PTSs during pancreatic cancer tumorigenesis, however, the exact mechanism has yet to be elucidated.

## Discussion

Few typical symptoms and signs in the early stage of the disease, rapid progression and a lack of effective early diagnostic and treatment options are the main clinical features of pancreatic cancer, leading to its poor prognosis [1, 2]^.^ Most pancreatic cancer patients are diagnosed in an advanced stage, which means that surgical treatment is not an option for them; however, adjuvant chemotherapy has limited efficacy [2, 3, 33, 34]. Hence, improving the early diagnosis of pancreatic cancer is the key to pancreatic cancer prevention and treatment, which requires a deeper understanding of the mechanisms of pancreatic cancer initiation. Using a KC transgenic mouse model, which can mimic the whole process of the initiation and progression of pancreatic cancer, especially from normal acinar cells to ADM and subsequent PanINs [14, 35], we demonstrated that the EGFR-HSF1axis promoted the initiation of pancreatic cancer.

Recent studies have suggested that PDAC may predominately originate from pancreatic “centro-acinar/acinar cells” [2, 36]. Driven by the *KRAS* oncogene mutation caused abnormal activation of multiple pathways (mainly on Ras-MAPK and PI3K-AKT-mTOR pathways [25]), the homeostasis of acinar cells was remolding [6] and the phenotype of pancreatic acinar cells gradually transforms from acinar cells to ductal-like cells (acinar cells undergo ADM); in this process, the transcriptome of acinar cells changed (the loss of acinar markers amylase and the gain of ductal markers CK19, [37]. Mentionable, HSF1/HSPs are important for the maintenance of the proteostasis of cells (including normal pancreatic acinar cells) in various stress condition and protect them against the damage of PTSs such as pancreatitis [29]. In this study, we found that EGFR-HSF1 axis was important for *Kras* oncogene mutation induced abnormal MAPK pathway activation driven PTSs and pancreatic cancer tumorigenesis. Nevertheless, targeting therapy on *KRAS* oncogene mutation seems unrealistic, so some alternative treatments targeting its downstream pathways (especially on breaking PTSs and restoring the proteostasis of acinar cells, such as modulating HSF1) need to be used to prevent the initiation of PDAC and improve prognosis of pancreatic cancer patients.

Our previous researchers found that the abnormal activation of HSF1 promoted the invasion and metastasis of pancreatic cancer, along with the observation that high-level HSF1 correlated with the poor prognosis of many cancers, including pancreatic cancer [7, 8, 38]. In this study, we detected that during the tumorigenesis process of pancreatic cancer, the activation form of HSF1 was accumulation in cytoplasm even early in ADM structures and then translocated into nucleus as the disease progress; when these mice suffered from acute/chronic pancreatitis, this process were significantly accelerated [39]. Besides, the pharmacological inhibition of HSF1 suppressed the formation of ADM and PanINs (in other words, HSF1 inhibition may suppress pancreatic cancer initiation in KC mice). Notably, for normal pancreatic acinar cells, plasticity is an important feature, which means that these cells can recover from ADM to assume normal acinar functions when the self/external pressure (such as pancreatitis and pancreas injury) are removed [40]; however, in the context of *Kras* oncogene mutation, regardless of whether there is a stimulus present or not, the fate of pancreatic acinar cells is set (irreversible ADM, subsequent PanINs and ultimately PDAC) [41]. Similarly, we found that only acinar edema remained in vehicle C mice pancreases when they recovered from pancreatitis; however, vehicle KC mice pancreases exhibited a large amount of pancreatitis-induced ADM, PanINs and even PDAC (along with an abundant desmoplastic reaction). Interestingly, after we treated mice with KRIBB11 to inhibit the activation of HSF1, the formation of pancreatitis-induced ADM, PanINs and PDAC was partly blocked in vivo, like the in vitro experiments with pancreatic acinar cell 3D culture. In conclusion, all above findings indicated that HSF1 and HSPs were important for the fate change of pancreatic acinar cells, in other words, the initiation of pancreatic cancer [42].

HSF1 supports tumorigenesis in various ways; for example, sustaining the proteostasis of cancer cells [6], modulating the metabolic pattern of tumor cells [43, 44], maintaining the maturation of cancer-related proteins through downstream chaperones [45], regulating pro-cancer signaling [46] and influencing the interaction between cancer cells and stromal cells [7, 47]. However, the abnormal activation alone HSF1 is not sufficient to cause cancer initiation [48], which means that the pro-tumorigenesis effect of HSF1 requires the contributions of other cancer-promoting mechanisms (such as the *KRAS* oncogene mutation). Apart from its critical role in cancer initiation, increasing evidence suggests that cancer cells become reliant on HSF1 to maintain their malignant phenotypes [48, 49]. Specifically, tumor cells express high levels of HSF1 via a variety of methods, for example, inhibiting the ubiquitinated degradation of HSF1 [50], reducing the expression of molecules that compete with HSF1 [51, 52] and stimulating HSF1 activation-related pathways (such as EGFR mediated MAPK pathway [6]).

In this study, we found a close relationship between HSF1 and EGFR pathway through bioinformatics analysis and the parallel activation between EGFR pathway and HSF1 in pancreatic precancerous lesions was also confirmed by through IHC staining. Considering that the abnormal activation of the normal EGFR pathway and its downstream MAPK/mTOR pathways followed by the *KRAS* oncogene mutation is essential to the initiation and progression of pancreatic cancer [53–55], these results remind us that EGFR-HSF1 axis might participate in the pancreatic cancer initiation efficiently. Hence, we carried out a series in vivo and in vitro studies, and results showed that the EGFR activation mediated pancreatic cancer initiation was partly HSF1 dependent. Besides, we also found EGFR and its downstream MEK/mTOR closely participated the active-phosphorylation and nuclear-translocation of HSF1 in pancreatic acinar cells; interestingly, the EGFR-Ras-MAPK pathway may activate HSF1 via induce the PTSs of pancreatic acinar cells indirectly, in other words, the abnormal level of HSF1 during pancreatic cancer tumorigenesis may be a passive response of the cells against to the increasing PTSs. Mentionable, sustained PTSs is inevitable during the cancer initiation and progression [49], and studies have shown that cancer cells are more sensitive to PTSs and have a stronger demand for homeostasis than normal cells [6]; hence, the abnormal activation of HSF1 in cancer cells may emphasize the core role of HSF1 on maintain cell homeostasis during tumorigenesis and progression once again [56, 57].

However, pancreatic cancer tumorigenesis is a complete process. Studies have shown that the abnormal activation of the normal EGFR signaling pathway followed by the *Kras* oncogene mutation is essential to the initiation and progression of pancreatic cancer [58]; nevertheless, we found that a certain number of cells undergo malignant transformation even in the background of EGFR-HSF1 axis inhibition, which indicated the presence of other important components in the initiation of pancreatic cancer. In addition, both in vivo and in vitro, HSF1-mediated heat shock responses are key events that maintain cellular proteostasis during heat shock and other stress; thus, the normal physiological activities of cells were greatly affected when we entirely blocked the function of HSF1 (in other words, it may cause serious side effects in patients who undergo HSF1 inhibition treatment), and the optimal therapeutic strategy is to weaken the tumor-promoting effect of HSF1 while maintaining its heat shock response function [59]. Therefore, much research is necessary to overcome these issues.

## Conclusion

In conclusion, the present study demonstrated that HSF1 plays a crucial role in the initiation of pancreatic cancer and that EGFR and its downstream signaling pathway sustain the pro-tumorigenesis effect of HSF1. Nevertheless, the specific underlying mechanisms are complex and still need to be explored.

## Supplementary Information

**Additional file 1: Fig. S1.** Related technologies mentioned in this article and basic morphology/pathology features of KC mice. **Fig. S2.** Pharmacological inhibition of HSF1 suppressed the formation of ADM in vitro. **Fig. S3.** EGFR stimulation activated HSF1 doubly in pancreatic acinar cells.

**Additional file 2: Table S1.** Source files of bioinformatics analysis on HSF1 and its related proteins (mainly TCGA PAAD datasets).

**Additional file 3: Table S2.** Source files of bioinformatics analysis on EGFR (mainly TCGA PAAD datasets).

**Additional file 4: Table S3.** Source files of bioinformatics analysis on EGFR pathway (GEO GSE98399 datasets).

## Data Availability

All data generated or analyzed during this study are available from the corresponding author on reasonable request.

## Abbreviations

PDAC: pancreatic ductal adenocarcinoma
HSF1: heat shock factor 1
HSPs: heat shock proteins
HSRs: heat shock responses
HSSs: heat shock stress
PTSs: proteotoxic stress
EGFR: epidermal growth factor receptor
ADM: acinar-ductal metaplasia
PanINs: pancreatic intraepithelial neoplasias
KC: *LSL-Kras*^*G12D/+*^*; Pdx1-Cre*
AP-ADMs: acute pancreatitis-ADM models
CP-PanINs: chronic pancreatitis-PanINs models
UPR: unfolded protein reaction.
