# Supplementary material for: The EGFR-HSF1 axis accelerates the tumorigenesis of pancreatic cancer
Source: J Exp Clin Cancer Res. 2021 Jan 9;40:25. doi: 10.1186/s13046-020-01823-4 (PMC7797143; doi:10.1186/s13046-020-01823-4)
Supplement: Supplementary file 1 — Additional file 1: Fig. S1. Related technologies mentioned in this article and basic morphology/pathology features of KC mice. Fig. S2. Pharmacological inhibition of HSF1 suppressed the formation of ADM in vitro. Fig. S3. EGFR stimulation activated HSF1 doubly in pancreatic acinar cells. [file 13046_2020_1823_MOESM1_ESM.docx]

**Supplement figure and figure legends**

**
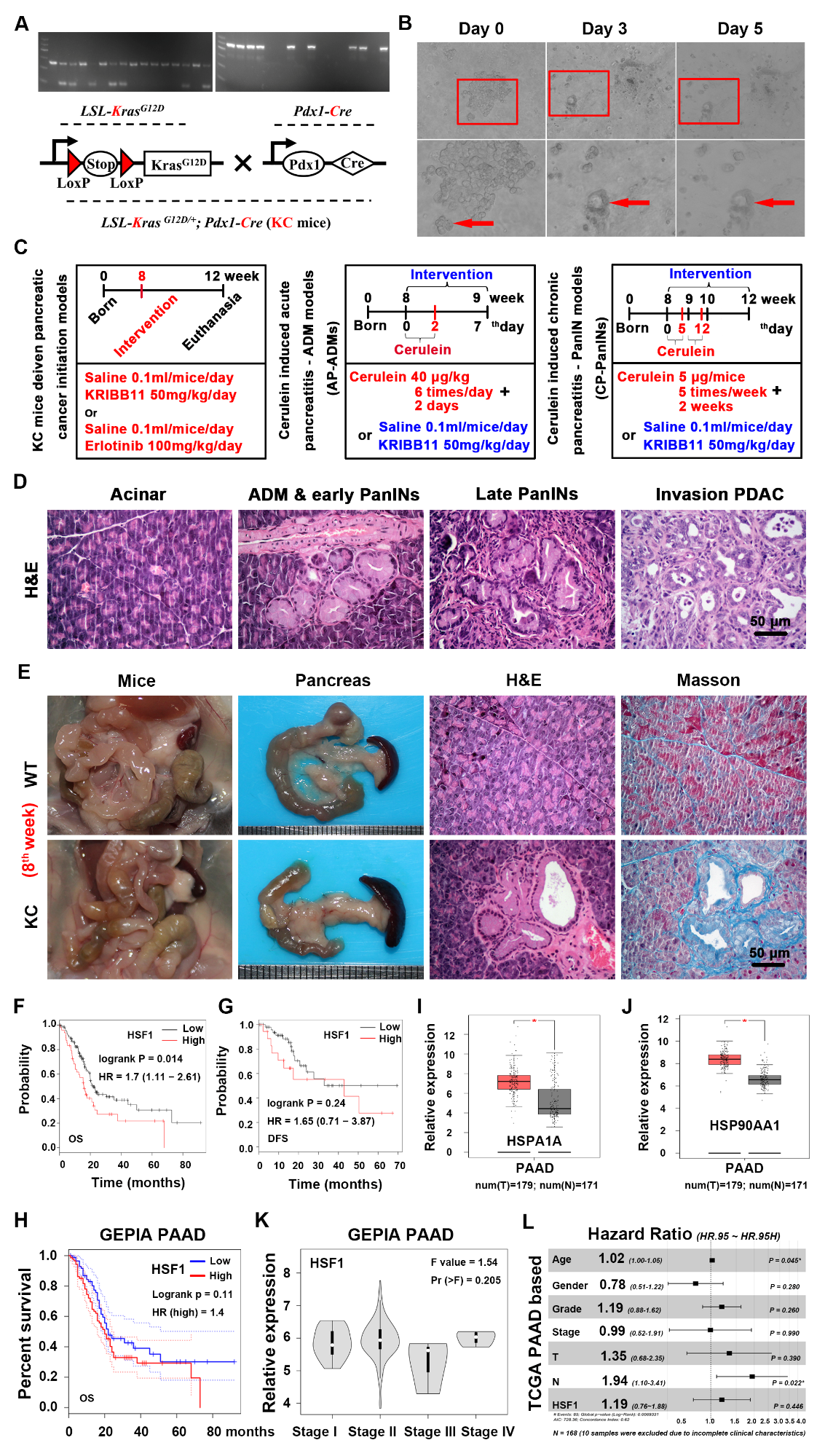
**

**Figure S1. Related technologies mentioned in this article and basic morphology/pathology features of KC mice. (A)** A brief mechanism diagram and PCR on illustrating the pattern of crossing to obtain KC mice. **(B)** Representative bright field images of pancreatic acinar 3D culture in day 0, 3 and 5. **(C)** Detailed intervention strategies on the initiation models (left part), ADM-acute pancreatitis models (middle part) and PanINs-chronic pancreatitis models (right part). **(D)** Representative histopathology (display by H&E staining) of all stages of the tumorigenesis of pancreatic cancer in KC mice. **(E)** Representative morphology, histopathology (display by H&E staining) and fibrosis (display by Masson staining) of 8-weeks-old KC mice. **(F-G)** The relationship between HSF1 and the overall survival (F, OS)/disease free survival (G, DFS) of pancreatic patients according to online websites (Kaplan-Meier Plotter). **(H)** The relationship between HSF1 and the OS of pancreatic cancer patients according to online websites (GEPIA). **(I-J)** Relative HSPA1A (I) and HSP90AA1 (J) expression in normal pancreas tissues and pancreatic cancer tissues according to online websites (GEPIA). **(K)** The relative HSF1 mRNA expression in four clinical stage of pancreatic cancer according to online website (GEPIA). **(L)** The forest graph showed Hazard Ratio (HR) and its 95% Confidence Interval (CI) of HSF1 in pancreatic cancer patients’ OS according to TCGA database. Scale bars = 50 μm. ***** P < 0.05.


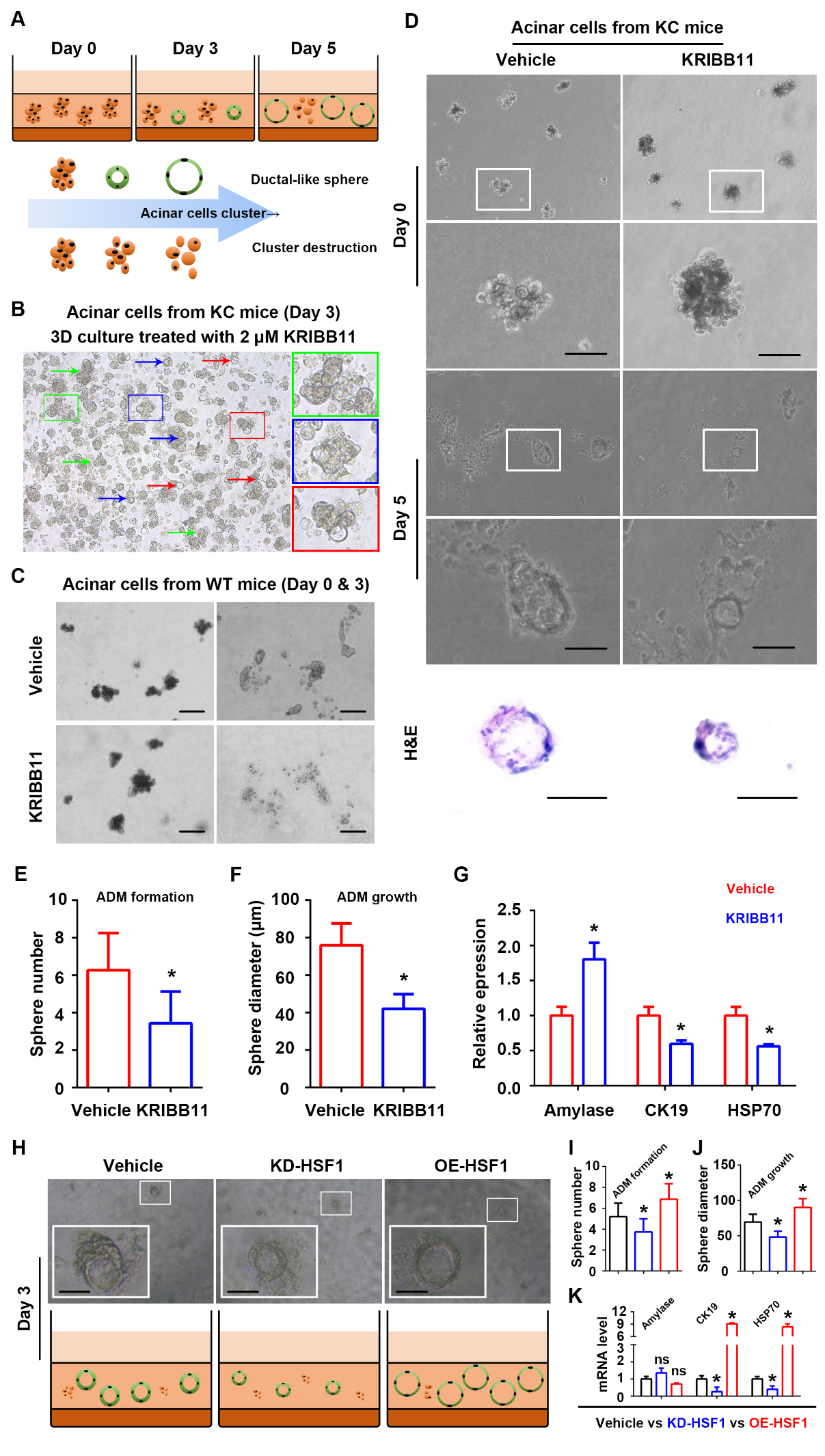


**Figure S2.** **Pharmacological inhibition of HSF1** **suppressed** **the formation of ADM in vitro.** **(A)** A brief mechanism diagram on illustrating pancreatic acinar 3D culture in day 0, 3 and 5. **(B)** Representative bright field images of 3D acinar treated with 10 μM KRIBB11 for 3 days (acinar from KC mice, green boxes and arrows represent phenotypic normal acinar cells, blue boxes and arrows represent acinar cells with a phenotype that are undergoing ADM, red boxes and arrows represent ductal-like sphere). **(C-D)** Representative bright field/H&E staining images of 3D acinar/ductal-like sphere between vehicle and KRIBB11 group (acinar from WT mice (C) and acinar cell form KC mice (D)). **(E-F)** Quantification of the sphere number (E) and diameter (F) per 100× field between vehicle and KRIBB11 group (acinar from KC mice). **(G)** Relative amylase, CK19 and HSP70 mRNA level of sphere among vehicle and KRIBB11 group (acinar from KC mice). **(H)** Representative bright field images of 3D acinar/ductal-like sphere between vehicle lentiviral vector, HSF1 knock-down lentiviral vector (KD-HSF1) and HSF1 over-expression lentiviral vector (OE-HSF1) group (acinar from KC mice). **(I-J)** Quantification of the sphere number (I) and diameter (J) per 100× field between vehicle, KD-HSF1 and OE-HSF1 group (acinar from KC mice) **(K)** Relative amylase, CK19 and HSP70 mRNA level of sphere among vehicle, KD-HSF1 and OE-HSF1 group (acinar from KC mice, the vehicle group was a control). Scale bars = 50 μm. ***** P < 0.05.


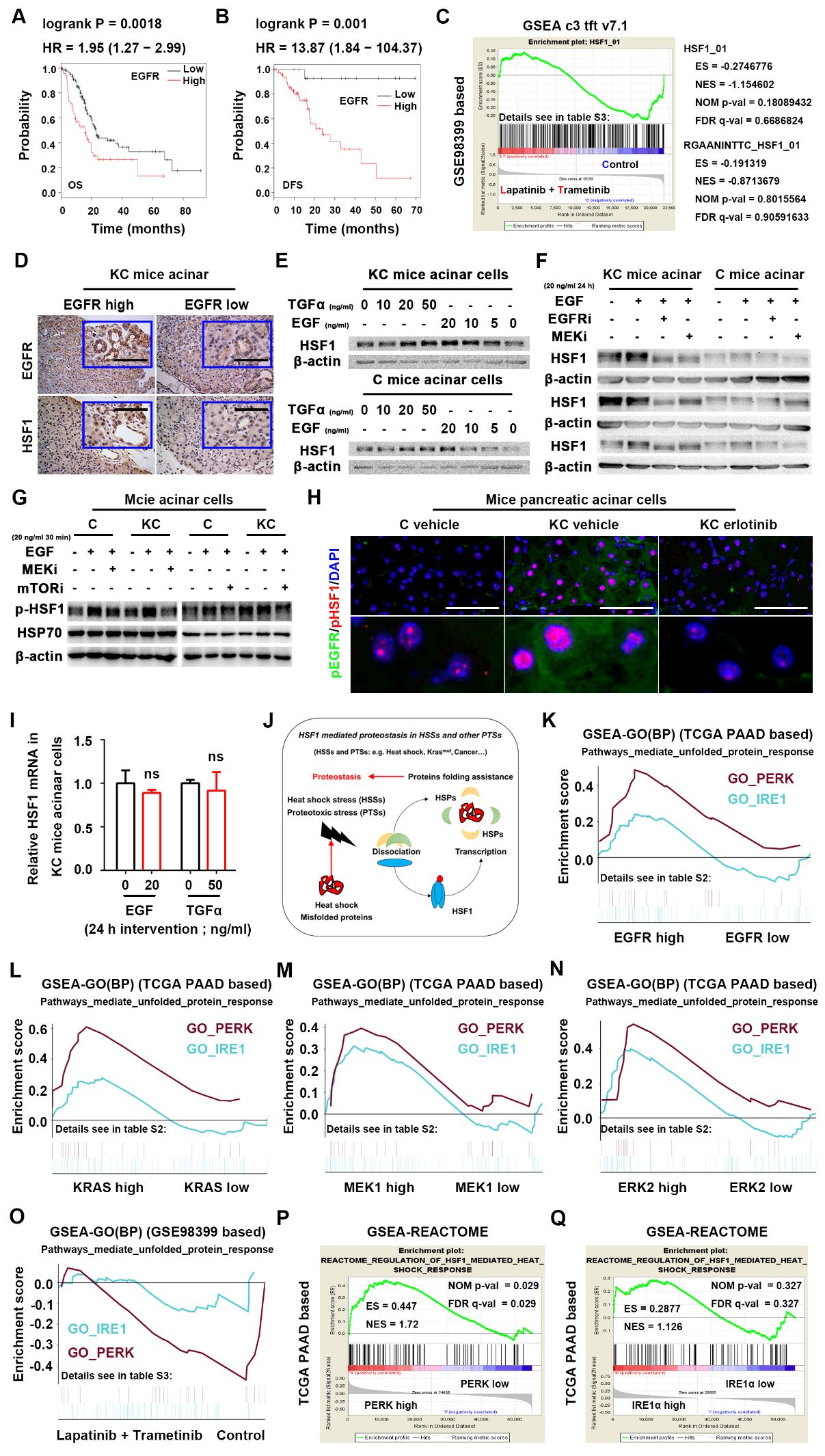


**Figure S3: EGFR stimulation activated HSF1 doubly in pancreatic acinar cells. (A-B)** The relationship between EGFR expression and pancreatic patients’ OS (A)/DFS (B) according to online websites (Kaplan-Meier Plotter). **(C)** GSEA analysis of HSF1 binding motifs/signature (target genes gene sets, represented by RGAANNTTC_V$HSF1_01 and HSF1_01) in control (acute vehicle control treatment) group vs intervention (acute trametinib plus lapatinib treatment) group according to the GSE98399 datasets. **(D)** Representative IHC staining of EGFR and HSF1 in the pancreas of KC mice and C mice. **(E)** Western blotting was performed to evaluate the expression of HSF1 in KC/C mice acinar cells in the background of 24 hours EGF or TGFα exposure. **(F)** Western blotting was performed to evaluate the expression of total HSF1 in KC mice acinar cells in the background of 24 hours EGF exposure, or EGF plus erlotinib (EGFRi)/selumetinib (MEKi). **(G)** Western blotting was performed to evaluate the S326 p-HSF1 in mice acinar cells in the background of 30 min EGF exposure, or EGF plus selumetinib (MEKi)/torkinib (mTORi). **(H)** Double-label IF staining showed the expression of Y1068 p-EGFR (green) and S326 p-HSF1 (red) in the acinar of vehicle C mice, vehicle KC mice and KC mice treated with erlotinib. **(I)** qRT-PCR was performed to evaluate the expression of HSF1 in KC/C mice acinar cells in the background of 24 hours EGF or TGFα exposure (0 μM group was a control). **(J)** The diagram of HSF1 activation mediated HSRs in HSSs and other PTSs. **(K-N)** GSEA analysis of two UPR pathways (IRE1α/PERK) in EGFR/KRAS/MEK1/ERK2 high expression group vs EGFR/KRAS/MEK1/ERK2 low expression group according to a TCGA datasets of pancreatic cancer. **(O)** GSEA analysis of two UPR pathways (IRE1α/PERK) in control (acute vehicle control treatment) group vs intervention (acute trametinib plus lapatinib treatment) group according to the GSE98399 datasets. **(P-Q)** GSEA analysis of HSF1 activity in PERK/IRE1α high expression group vs PERK/IRE1α low expression group according to a TCGA datasets of pancreatic cancer. Scale bars = 50 μm. ns P > 0.05, ***** P < 0.05.
